# Prevalence and Antimicrobial Susceptibility of Foodborne Pathogens from Raw Livestock Meat in China, 2021

**DOI:** 10.3390/microorganisms12112157

**Published:** 2024-10-26

**Authors:** Xiang Ren, Dajin Yang, Zushun Yang, Ying Li, Shuran Yang, Weiwei Li, Xin Qiao, Chengyu Xue, Min Chen, Limin Zhang, Lin Yan, Zixin Peng

**Affiliations:** 1 NHC Key Laboratory of Food Safety Risk Assessment, Chinese Academy of Medical Science Research Unit (2019RU014), China National Center for Food Safety Risk Assessment, Beijing 100022, China; kmrxhb@hotmail.com (X.R.); yangdajin@cfsa.net.cn (D.Y.); liying@cfsa.net.cn (Y.L.); yangshuran@cfsa.net.cn (S.Y.); weiweili@cfsa.net.cn (W.L.); 2 Yunnan Provincial Center for Disease Control and Prevention, Kunming 650000, China; yzsylc@126.com (Z.Y.); chenminyx@hotmail.com (M.C.); kmzlmhb@hotmail.com (L.Z.); 3 Jiangsu Provincial Center for Disease Control and Prevention, Nanjing 210009, China; jscdcqx@126.com; 4 Heilongjiang Provincial Center for Disease Control and Prevention, Harbin 150030, China; 15046060902@163.com

**Keywords:** *Listeria monocytogenes*, *Salmonella*, diarrheagenic *Escherichia coli*, raw livestock meat, prevalence, serovar, antimicrobial resistance

## Abstract

The rising prevalence of pathogenic bacteria in livestock meat poses a growing public health concern in China. The determination of antimicrobial resistance (AMR) is critical for the clinical management of foodborne infections stemming from livestock meat consumption. This study aimed to assess the prevalence of pathogenic bacteria in livestock meat (pork, beef, and mutton) sampled in China in 2021 and to identify the most common AMR patterns among the isolated pathogens. A total of 2515 raw livestock meat samples were collected across 15 provinces in China during 2021. Pathogen detection, including *Listeria monocytogenes*, *Salmonella*, and diarrheagenic *Escherichia coli* (DEC), followed China’s national food safety standards. All *Salmonella* isolates underwent serotyping via slide agglutination. Antimicrobial susceptibility of *Salmonella* and DEC isolates was assessed using the broth dilution method. The detection rates for *L. monocytogenes*, *Salmonella*, and DEC in raw livestock meat were 9.06% (228/2, 515), 10.54% (265/2, 515), and 6.16% (155/2, 515), respectively. Pork showed the highest contamination rates for *Salmonella* and DEC, with prevalence rates of 17.60% (214/1, 216, χ2 = 124.62, *p* < 0.05) and 7.89% (96/1, 216, χ2 = 14.466, *p* < 0.05), respectively. *L. monocytogenes* contamination was notably higher in chilled (14.43%, 84/582) and frozen (12.39%, 55/444) meat than in fresh meat (χ2 = 43.510, *p* < 0.05). In contrast, *Salmonella* (12.09%, 180/1489, χ2 = 15.173, *p* < 0.05) and DEC (7.25%, 108/1489, χ2 = 12.275, *p* < 0.05) were more prevalent in fresh meat than in chilled or frozen samples. The predominant *Salmonella* serotypes identified were *Salmonella enterica* subsp. *enterica* serovar Typhimurium, followed by *Salmonella enterica* serovar Derby, *Salmonella enterica* serovar Rissen, *Salmonella enterica* serovar London, and *Salmonella enterica* serotype Enteritidis. Enteroaggregative *E. coli* was the most frequent pathotype among DEC (84.7%, 133/157), followed by enteropathogenic *E. coli* (8.3%, 13/157) and enterohemorrhagic *E. coli* (5.1%, 8/157). Among the 14 tested antimicrobial agents, *Salmonella* isolates demonstrated an overall resistance rate of 87.50%, while DEC exhibited a resistance rate of 84.70%. Ampicillin and tetracycline showed the highest resistance rates in both pathogens. Multi-drug resistance (MDR) was observed in 67.53% of *Salmonella* isolates (183 isolates) and 57.96% of DEC isolates (91 isolates). This study highlights the significant contamination of retail raw livestock meat in China by *L. monocytogenes*, *Salmonella*, and DEC. The high resistance of MDR in both pathogens poses serious public health risks. Chinese food safety and veterinary authorities should implement stricter measures to control pathogen contamination and regulate the use of antimicrobials in livestock to mitigate these risks.

## 1. Introduction

Foodborne diseases are a major global public health issue, leading to significant consumption of healthcare resources and socio-economic losses [1,2]. The World Health Organization (WHO) reports that approximately 600 million people suffer from foodborne illnesses annually, with nearly 420,000 deaths worldwide [3]. Between 1991 and 2021, 1729 outbreaks of foodborne illness linked to livestock meat consumption resulted in 41,438 infections and 10,063 deaths globally [4]. Beef, pork, and mixed meats are the primary sources of these infections and outbreaks [5,6,7,8]. The most common pathogens responsible for foodborne outbreaks related to livestock meat are *Salmonella*, diarrheagenic *Escherichia coli*, and *Listeria monocytogenes* [9].

*L. monocytogenes* is known to cause listeriosis, a serious condition associated with diarrhea, severe infections, sepsis, meningitis, and even miscarriages. The mortality rate of listeriosis is high, 20% to 30% in vulnerable patients, such as the elderly, immunocompromised patients, pregnant women, and infants [10]. According to the Centers for Disease Control (CDC, Atlanta, GA, USA), approximately 1600 people get listeriosis each year, with approximately 260 people dying from the disease [11]. This pathogen exhibits remarkable resilience and is capable of surviving under extreme conditions, such as low temperatures, acidic environments, and high salt concentrations—conditions typical in food processing facilities. Furthermore, the ability of *L. monocytogenes* to form biofilms enables it to persist for years in processing plants, highlighting the critical need for continuous monitoring of this pathogen in food products [12].

*Salmonella* is another leading cause of foodborne illness, contributing significantly to global diarrheal diseases. The WHO estimates that *Salmonella* is responsible for 94 million cases of gastroenteritis and 33 million disability-adjusted life years (DALYs) lost each year. Non-typhoidal *Salmonella enterica* accounts for 4.07 million of those DALYs, representing the most substantial disease burden [13]. In China, *Salmonella* is particularly concerning, and it is associated with 70–80% of bacterial food poisoning cases [14]. The risk of *Salmonella* contamination is present at all stages of pork production, from processing to retail [15]. Raw pork is especially vulnerable, and given that China is the largest pork consumer in the world, the risk of contamination in pork production is notably higher than that in other countries [16].

DEC is an important foodborne pathogen that has been classified into six pathogenic types on the basis of their specific virulence traits. These types include enteropathogenic *E. coli* (EPEC), enterotoxigenic *E. coli* (ETEC), enteroaggregative *E. coli* (EAEC), enteroinvasive *E. coli* (EIEC), diffusely adherent *E. coli* (DAEC), and enterohemorrhagic *E. coli* (EHEC). This pathogen can acquire genes from external sources that encode virulence factors and antimicrobial resistance genes, enabling it to adapt to various environments. It is commonly found in a variety of food sources, including raw poultry, beef, and pork [17,18]. It is typically transmitted to humans through contaminated food, making it a significant indicator of meat safety [19,20]. Monitoring data from the United States in 2015 showed that 5.0% of fresh pork was contaminated with DEC [21]. Meanwhile, monitoring data from the European Union in 2017 showed that the contamination rate of DEC in fresh pork was 4.4% [9]. Even though the rate is not high overall, the strains, especially EAEC, EPEC, and ETEC, are responsible for around 30–40% of diarrhea episodes in children aged less than five years [22]. Diarrhea caused more than 1.3 million young children’s deaths worldwide in 2013 [23], which increased to 1.6 million cases annually in 2016 [24]. In 2019, over 1300 young children died because of diarrhea each day worldwide [25]. The efficacy of antibiotics as a treatment for diarrhea has been increasingly limited by irregular and uncontrolled self-prescription of antibiotics in developing countries in recent decades. Many first-line drugs such as ampicillin, ciprofloxacin, and sulfamethoxazole/trimethoprim are now limited by the ineffectiveness [26]. DEC is adaptable and is coupled with resistance to antibiotics and sanitizers commonly used in the food industry, which makes it a substantial threat to human health [27]. Therefore, monitoring the pathogenicity and contamination rates of DEC in food is essential for ensuring public health safety [28]. In China, enteropathogenic *E. coli* (EPEC) is the most prevalent diarrheagenic strain causing foodborne illness [29,30].

Meat serves as an ideal culture medium for microorganisms and is highly susceptible to contamination by various pathogenic bacteria. Several studies have indicated that meat and meat products are the leading causes of foodborne diseases in China, accounting for 28.2% of cases of human infections [31,32]. As meat consumption increases due to shifting dietary habits in China, the incidence of foodborne diseases and related public health concerns continues to rise [33].

The aim of this study was to assess the prevalence and AMR profiles of key foodborne pathogens in retail raw livestock meat from 15 provinces in China. These findings provide critical insights into the contamination status of pathogenic bacteria and contribute to a better understanding of microbial food safety in raw livestock meat, thereby supporting efforts to improve food safety controls from farm to table.

## 2. Materials and Methods

### 2.1. Sample Collection

**Figure 1:**
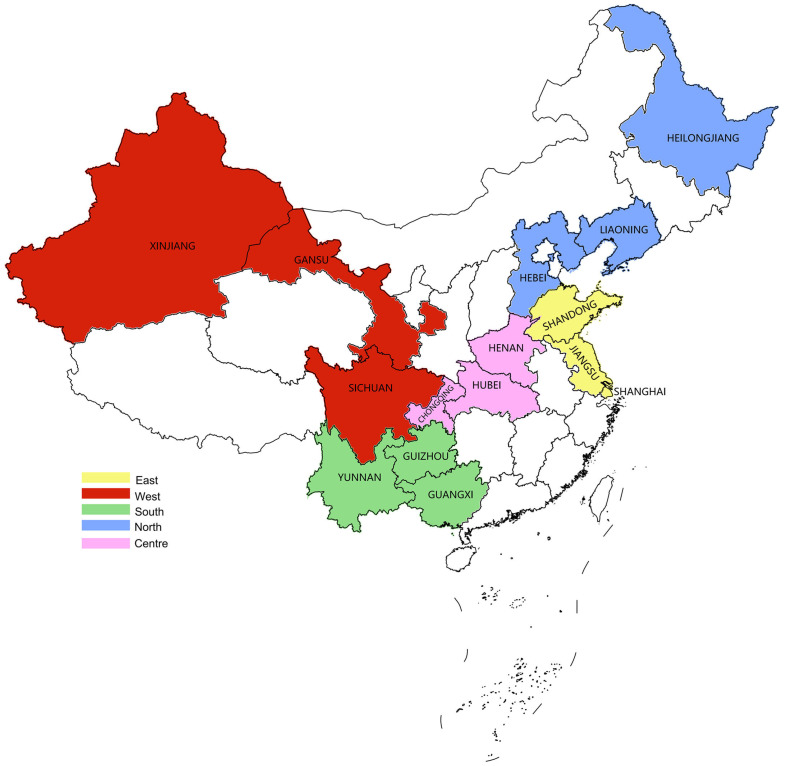
Sampling provinces or cities and regions in China.

In 2021, a total of 2515 raw livestock meat samples were collected from 15 provinces across five regions of China: the Eastern region, Southern region, Western region, Northern region, and Central region (Figure 1). The provinces investigated were Shanghai, Jiangsu, and Shandong in the Eastern region; Guangxi, Guizhou, and Yunnan in the Southern region; Sichuan, Gansu, and Xinjiang Autonomous Region in the Western region; Hebei, Heilongjiang, and Liaoning in the Northern region; and Henan, Hubei, and Chongqing in the Central region. Samples were randomly collected from farmers’ markets, supermarkets, retail shops, and catering establishments. The collection included pork (*n* = 1216), beef (*n* = 779), and mutton (*n* = 520) samples, which encompassed fresh (*n* = 444), chilled (*n* = 582), and frozen (*n* = 1489) meats. To assess the impact of different quarters on the contamination rates of pathogenic bacteria, the survey was conducted throughout all four quarters of the year. Sampling procedures adhered to the national standard for meat and meat product sampling, as specified in GB/T 4789.17-2003 [34]. Detailed sample information is provided in Table 1.

### 2.2. Isolation and Identification of Pathogens

The detection of *L. monocytogenes*, *Salmonella*, and DEC was carried out in accordance with the China National Food Safety Standards: GB 4789.30-2016 [35], GB 4789.4-2016 [36], and GB 4789.6-2016 [37], respectively. For *L. monocytogenes*, 25 g livestock meat samples are homogenized and incubated in 225 mL of Listeria Enrichment Broth I culture (Beijing Landbridge Technology Co., Ltd., Beijing, China, LB1) for 24 h. Then, LB1 enrichment culture is added to Listeria Enrichment Broth II culture (Beijing Landbridge Technology Co., Ltd., Beijing, China, LB2) for a second 24 h enrichment. Next, Listeria Chromogenic Agar (Beijing Landbridge Technology Co., Ltd., Beijing, China) and PALCAM (Beijing Landbridge Technology Co., Ltd., Beijing, China) can be used together to isolate presumptive colonies, which can be purified on tryptic soy agar with yeast extract and identified via Gram staining and biochemical confirmation.

For *Salmonella*, the specific steps were as follows. Each sample of 25 g of livestock meat was placed in a separate sterile shaker flask and washed with 225 mL of buffered peptone water with vigorous shaking for 5 min. The rinse was incubated at 37 °C in a water bath with shaking at 200 rpm for 8 h, and then 10 mL of buffered peptone water was added to 100 mL of selenite cystine broth at 37 °C for 16 to 24 h. A loop of inoculum from the selenite cystine broth was streaked onto Salmonella-Shigella agar (Oxoid Ltd., Basingstoke, UK, SS) or xylose lysine deoxycholate agar (Oxoid Ltd., Basingstoke, UK, XLD) and incubated for 16 to 24 h at 37 °C. Presumptive strains were picked from each plate by testing in triple sugar iron and lysine iron agar slants and incubated for 16 to 24 h at 36 °C. Isolates with typical *Salmonella* phenotypes were confirmed using API 20E test strips (bioMerieux Vitek, Marcyl’Etoile, France). Confirmed *Salmonella* isolates were serotyped using the slide agglutination method with commercial O and H antisera (Statens Serum Institut, Copenhagen S, Denmark, SSI), and the results were interpreted following the Kauffmann–White scheme.

For DEC, 25 g livestock meat samples are homogenized and incubated in 225 mL nutrient broth for 6 h at 37 °C. Then, 10 μL of nutrient broth was added to 30 mL of EC broth enriched at 42 °C for 18 h. A loop of inoculum from the EC broth was streaked onto MacConkey agar (MAC, Oxoid Ltd., Basingstoke, UK) and Eosin Methylene Blue (EMB, Oxoid Ltd., Basingstoke, UK) and incubated for 16 to 24 h at 37 °C. Ten putative DEC colonies were confirmed using API 20E test strips (bioMerieux Vitek, Marcyl’Etoile, France). DEC pathotypes were identified by the PCR method. The colonies were then selected and mixed with 150 μL water to extract DNA at 100 °C for 10 min. The 20 μL volume of quantitative PCR (qPCR) mix was composed of 10 μL master mix (Takara Bio Inc., Shlga, Japan), 1 μL forward primer (10 μmol), 1 μL reverse primer (10 μmol) [37], 1 μL DNA template, and 7 μL H_2_O. The cycling conditions for each subtype DEC were 94 °C for 5 min, 30 cycles of 94 °C for 30 s, 63 °C for 30 s, extension at 72 °C for 1.5 min, and a final extension at 72 °C for 5 min. The subtypes of EAEC, EPEC, EHEC, and ETEC were determined by screening the *aggR*, *astA*, and *pic* genes, the *bfpB*, *escV*, *stx1*, and *stx2* genes, the *escV*, *stx1*, *stx2*, and *bfpB* genes, and the *lt*, *stp*, and *sth* genes.

### 2.3. AMR Testing

As evidenced by the existing literature and our own prior research findings, the resistance rate of *L. monocytogenes* in food is relatively low, whereas the resistance rates of *Salmonella* and DEC are considerably higher. Due to the limitations in funding, this project initially focused on resistance research on *Salmonella* and DEC with the highest resistance rates. Antibiotic susceptibility testing (AST) for *Salmonella* and DEC isolates was performed by determining the minimal inhibitory concentration values using the broth microdilution method, following the Clinical and Laboratory Standards Institute (CLSI, 2021) guidelines [38]. The following 14 antibiotics were tested: Quinolone antibiotics: ciprofloxacin (CIP) and nalidixic acid (NA); Chloramphenicol antibiotics: chloramphenicol (CHL); Aminoglycoside antibiotic: gentamicin (GEN); Tetracycline antibiotic: tetracycline (TET); Alkylating agents: cyclophosphamide (CTX); Cephalomycin: cefoxitin (CFX); Penicillin antibiotic: ampicillin (AMP) and ampicillin-sulbactam (AMS); Cephalosporins: ceftazidime (CAZ), cefazolin (CFZ), and Carbapenems: imipenem (IPM); Sulfonamide: trimethoprim-sulfamethoxazole (SXT); and Polymyxin: colistin (CT). Quality control strains, *E. coli* ATCC25922 and *Staphylococcus aureus* ATCC29213, were used throughout the testing. Isolates showing resistance to at least one antibiotic were classified as antimicrobial resistant, while those resistant to three or more antibiotics were defined as multi-drug-resistant (MDR) isolates.

### 2.4. Statistical Analysis

All statistical analyses were performed using SPSS 26.0 software (IBM Corp., Armonk, NY, USA) [39]. The *chi*-square test and non-parametric Kruskal–Wallis test were used to analyze the differences in the prevalence of *L. monocytogenes*, *Salmonella*, and DEC across different food categories, quarters, and geographical regions. A *p* value of < 0.05 was considered statistically significant.

## 3. Results

### 3.1. Prevalence of L. monocytogenes, Salmonella, and DEC in Raw Livestock Meat

The overall detection rates for *L. monocytogenes*, *Salmonella*, and DEC in the collected livestock meat samples were 9.06% (228/2515), 10.54% (265/2515), and 6.16% (155/2515), respectively. Detection rates varied across different locations, storage conditions, sample types, and quarters, as shown in Table 2. There was a highly significant difference (*p* < 0.05) in the prevalence of *Salmonella* and DEC among different meat types. The pork had the highest prevalence, with *Salmonella* detected in 17.60% (214/1216) of samples and DEC in 7.89% (96/1216). These rates were two to three times higher than those found in beef and mutton. However, no significant difference was observed in the prevalence of *L. monocytogenes* across different types of meat. The storage condition of the meat samples also had a highly significant impact on pathogen prevalence (*p* < 0.05). The detection rate of *L. monocytogenes* was notably higher in chilled (14.43%, 84/582) and frozen (12.39%, 55/444) meat than in fresh meat (5.98%, 89/1489) (*p* < 0.05). On the other hand, *Salmonella* (12.09%, 180/1489) and DEC (7.25%, 108/1489) were more prevalent in fresh meat than in chilled or frozen samples (*p* < 0.05).

Significant regional disparities in pathogen prevalence were observed (*p* < 0.05). *L. monocytogenes* (12.50%, 70/560) and DEC (12.68%, 71/560) were more prevalent in the Northern region. In contrast, in the Eastern region, only *L. monocytogenes* showed a relatively high prevalence rate of 11.02% (71/644), while DEC had the lowest prevalence rate across all regions, at 2.17% (14/644), compared with the other regions. Conversely, the highest prevalence rates of *Salmonella* were found in the Southern (14.85%, 56/377) and Western (13.49%, 68/504) regions. The Western region also had the lowest prevalence rates of *L. monocytogenes* (4.96%, 25/504) and DEC (2.58%, 13/504) (*p* < 0.05). The Central region showed a moderate prevalence of pathogenic bacteria, with DEC detected in 10.47% (45/430) of samples, ranking second among all surveilled regions.

Statistical analysis revealed significant variation in the detection rates of *L. monocytogenes* and *Salmonella,* depending on the sampling site. The highest prevalence of *L. monocytogenes* was found in samples obtained from supermarkets (12.40%, 78/629) (*p* < 0.05), while *Salmonella* prevalence was highest in samples from retail stores, at 12.05% (44/365) (*p* < 0.05). Samples collected from catering services showed relatively low prevalence rates for all three pathogens.

No statistically significant differences were found in pathogen prevalence between frozen and chilled meats across different quarters. However, the detection rate of DEC significantly increased in fresh meat samples collected during the third and fourth quarters. In contrast, the lowest prevalence of *Salmonella* was observed in the first quarter compared with other quarters. The prevalence of *L. monocytogenes* remained unaffected by seasonal variations.

### 3.2. Serotypes and AMR of Salmonella in Raw Livestock Meat

#### 3.2.1. Serotypes of *Salmonella*

**Figure 2:**
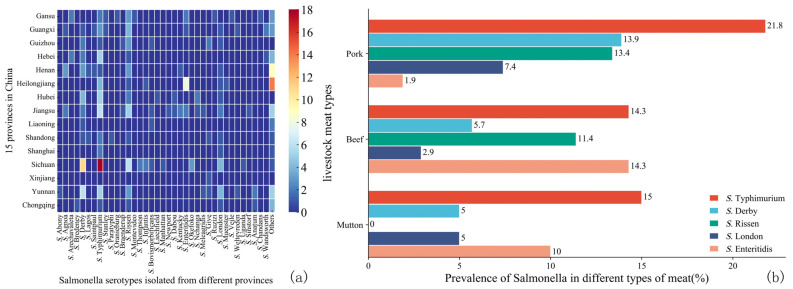
Distribution of various major serotypes of *Salmonella* isolated across different provinces and meat categories. (**a**) Heatmap of diverse *Salmonella* serotypes isolated from raw livestock meat in 15 provinces. (**b**) Proportional distribution of five major *Salmonella* serotypes among isolates from three categories of raw livestock meat (pork, beef, mutton).

A total of 271 *Salmonella* strains were isolated from 265 positive samples, with two different serotypes recovered from six samples simultaneously. The predominant serotype was *Salmonella enterica* serovar Typhimurium, accounting for 20.3% (55/271) of all isolates. Additionally, 33 isolates (12.2%) were identified as *Salmonella enterica subspecies enterica* serovar Derby and *Salmonella enterica* serovar Rissen, followed by *Salmonella enterica* serovar London (6.6%, 18/271) and *Salmonella* Enteritidis (4.8%, 13/271). As shown in Figure 2a, there were no notable differences in the distribution of *Salmonella* serotypes across the provinces. However, *S*. Typhimurium and *S*. Derby were most frequently detected in Sichuan Province, while *S.* Enteritidis was dominant in Heilongjiang Province.

Across different meat types, *S*. Typhimurium was the most prevalent serotype in pork, beef, and mutton (Figure 2b). In pork, the dominant serotypes were *S*. Typhimurium, *S*. Derby, *S*. Rissen, and *S*. London. In beef, *S*. Typhimurium, *S*. Rissen, and *S*. Enteritidis were common, while in mutton, *S*. Agona, *S*. Typhimurium, and *S*. Rissen were the most prevalent.

#### 3.2.2. AMR of *Salmonella*

**Figure 3:**
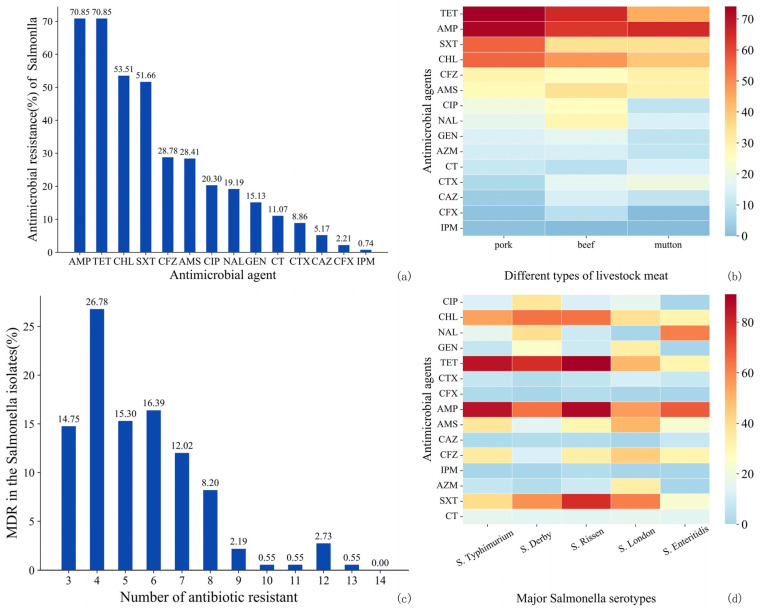
Antimicrobial resistance (AMR) of *Salmonella* isolated from raw livestock meat. (**a**) The AMR of 271 *Salmonella* isolates to 14 kinds of antibiotics. (**b**) Heat map of AMR rates of *Salmonella* isolated from different raw livestock meat (pork, beef, and mutton). (**c**) Multi-drug-resistant distribution in *Salmonella* isolates. (**d**) Heat map of resistance rates of five major *Salmonella* serotypes to 14 antibiotics.

The overall resistance rate of *Salmonella* isolates was 87.50%. More than 50% of the isolates demonstrated resistance to AMP (70.85%), TET (70.85%), CHL (53.51%), and SXT (51.66%) (Figure 3a). Conversely, the isolates exhibited the highest sensitivity to CTX (8.86%), CAZ (5.17%), CFX (2.21%), and IMP (0.74%). The *Salmonella* isolates from pork, beef, and mutton samples primarily displayed resistance to AMP, TET, CHL, and SXT (Figure 3b). Notably, the mutton *Salmonella* isolates showed the highest resistance to TET (63%). In contrast, pork *Salmonella* isolates had the lowest resistance rates to CTX (6.48%), CAZ (3.24%), CFX (1.39%), and IMP (0.93%). Beef *Salmonella* isolates exhibited the lowest resistance to azithromycin (AZM) (14.29%), CAZ (14.29%), CFX (8.57%), and CT (8.57%), with all isolates being sensitive to IPM.

A total of 183 *Salmonella* isolates (67.53%) displayed MDR phenotypes (Figure 3c). Most of these isolates (26.78%) showed AMR to four antibiotics, while 14.75% were resistant to more than eight antibiotics, with only one isolate demonstrating resistance to thirteen antibiotics. The pork *Salmonella* isolates exhibited the highest percentage of MDR (70.40%), followed by mutton (60.00%) and beef (54.30%).

The relationship between the main *Salmonella* serotypes and their AMR profiles is illustrated in Figure 3d. *S.* Typhimurium, *S.* Derby, and *S.* Rissen isolates exhibited the highest resistance to TET and AMP. *S.* London showed significant resistance to SXT and AMP, while *S.* Enterica demonstrated the highest resistance to AMP and NAL. Notably, *S.* London had a significantly higher resistance level to AMS (50.00%) than CHL (38.89%) (*p* < 0.05). Importantly, all three *Salmonella enterica* serovar Kentucky isolates showed MDR to 12 antibiotics.

### 3.3. Pathotypes and AMR of DEC in Raw Livestock Meat

#### 3.3.1. Pathotypes of DEC

Among the 157 isolates of DEC, the predominant pathotype was enteroaggregative *E. coli* (EAEC), accounting for 84.7% of the isolates. This was followed by EPEC at 8.3%, enterohemorrhagic *E. coli* (EHEC) at 5.1%, and enterotoxin-producing *E. coli* (ETEC) at 1.9%.

**Figure 4:**
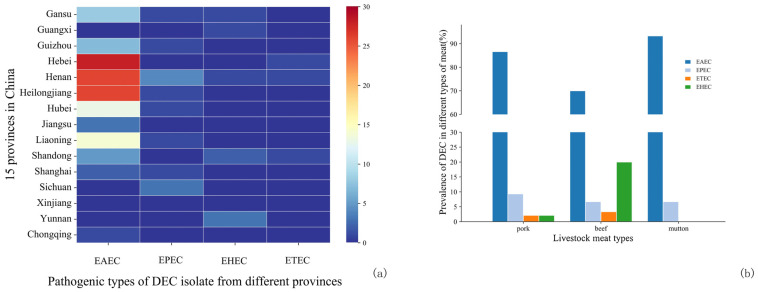
Distribution of various pathotypes of DEC isolated from 15 provinces or cities and different meat categories. (**a**) Heap map of different pathotypes of DEC isolated from raw livestock meat in 15 provinces. (**b**) The proportion distribution of four pathotypes in DEC isolates in each of the three categories of raw livestock meat (pork, beef, and mutton).

The prevalence of different pathogenic types of DEC varied by province (Figure 4). Henan, Hebei, and Heilongjiang Provinces exhibited the highest detection rates of EAEC, at 16.6% (26/157), 17.8% (28/157), and 16.6% (26/157), respectively. Additionally, Henan Province recorded a relatively high detection rate of EPEC at 2.5% (4/157), while Yunnan Province had a higher detection rate of EHEC at 1.9% (3/157).

The prevalence of different pathogenic types of DEC varied by province (Figure 4). Henan, Hebei, and Heilongjiang Provinces exhibited the highest detection rates of EAEC, at 16.6% (26/157), 17.8% (28/157), and 16.6% (26/157), respectively. Additionally, Henan Province recorded a relatively high detection rate of EPEC at 2.5% (4/157), while Yunnan Province had a higher detection rate of EHEC at 1.9% (3/157).

#### 3.3.2. AMR of DEC

**Figure 5:**
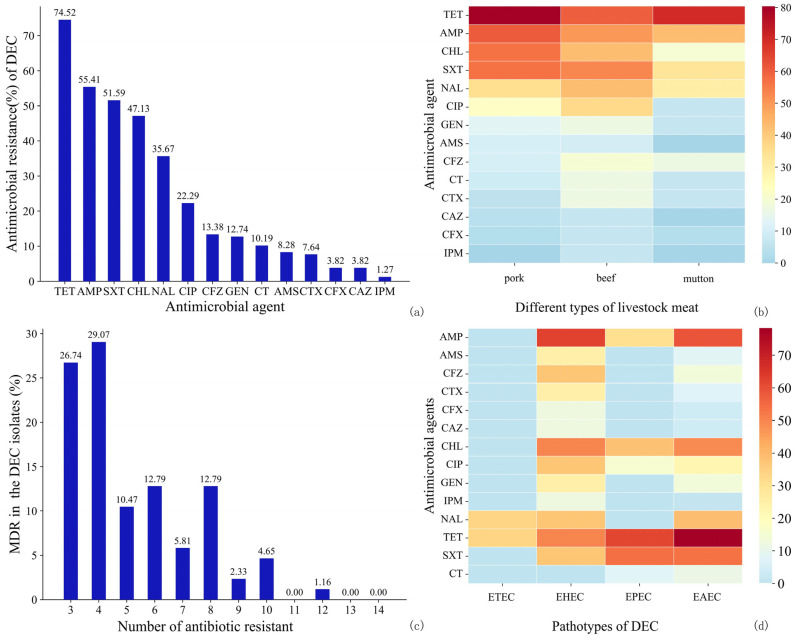
Antimicrobial resistance (AMR) of DEC isolated from raw livestock meat. (**a**) The AMR of 157 DEC isolates to 14 kinds of antibiotics. (**b**) Heat map of AMR rates of DEC isolated from different raw livestock meat (pork, beef, and mutton). (**c**) Multi-drug resistance (MDR) in DEC isolates. A total of 91 strains of DEC were MDR. (**d**) Heat map of resistance rates of 4 DEC pathotypes to 14 antibiotics.

The AMR profiles of DEC against 14 antibiotics are presented in Figure 5a. Overall, 84.70% of the isolates exhibited AMR. TET was identified as the most resistant antibiotic, with a resistance rate of 74.52%, followed by AMP at 55.41% and SXT at 51.59%. Conversely, all isolates displayed the highest sensitivity to CTX (92.36%), CFX (96.18%), CAZ (96.18%), and IPM (98.73%).

Pork-derived DEC isolates demonstrated the highest resistance to TET, AMP, SXT, and CHL while exhibiting the lowest resistance to CTX (5.15%), CAZ (4.12%), and CFX (3.09%). All pork isolates were sensitive to IPM (Figure 5b). In contrast, beef isolates displayed the highest resistance to TET (60.00%), SXT (53.33%), AMP (50.00%), CHL (43.33%), and NAL (43.33%), while showing the lowest resistance rates to AMS (10.00%), CAZ (6.67%), CTX (16.67%), and IPM (6.67%). Mutton isolates exhibited the highest resistance to TET (70.00%), AMP (43.33%), SXT (33.33%), and NAL (30.00%) but the lowest resistance rates to CIP (6.67%), GEN (6.67%), CT (6.67%), CTX (6.67%), and CFX (3.33%). Notably, all mutton isolates were resistant to AMS, CAZ, and IPM. The highest prevalence of MDR isolates was found in pork samples (67.00%), followed by beef (56.70%) and mutton (36.70%).

A total of 57.96% of the isolates were classified as MDR isolates (Figure 5c). Similar to the *Salmonella* isolates, the majority of DEC isolates exhibited resistance to four antibiotics. Only two isolates demonstrated resistance to 12 antibiotics, while none showed resistance to 11 antibiotics. Notably, four out of seven EHEC isolates were classified as MDR; among these, one isolate exhibited resistance to five antibiotics, whereas the remaining three showed resistance to more than eight antibiotics.

One ETEC isolate was sensitive to all tested antibiotics, while the other two showed resistance to TET and NAL, respectively. EHEC exhibited the highest resistance to AMP (62.50%), while EPEC was predominantly resistant to TET (61.54%). EAEC isolates demonstrated the greatest resistance to TET (78.20%) and AMP (58.65%).

## 4. Discussion

### 4.1. Prevalence of Foodborne Pathogens in Raw Livestock Meat

In this study, the prevalence of *L. monocytogenes* was found to be 9.06%, which is lower than the 16.8% reported in a European study spanning 21 years (2001–2022) [40]. Another study indicated that the prevalence of *L. monocytogenes* in raw meat in China was 11.8% between 2008 and 2016 [41]. In our previous study, the prevalence of *L. monocytogenes* in raw animal meat was recorded at 13.3% before 2010 and 9.8% after 2010, both of which were higher than the findings of the current study [42]. This decline may result from the gradual enhancement of institutional infrastructures and technical capabilities in China for monitoring and controlling *L. monocytogenes* throughout the food supply chain [43].

The prevalence of *L. monocytogenes* was 14.43% in chilled meat and 12.39% in frozen meat, and both are higher than that in fresh meat. This difference may be attributed to the pathogen’s ability to thrive in cold temperatures [44]. In our study, the detection rates of *L. monocytogenes* in supermarkets and retail stores were higher than those in farmers’ markets and catering establishments, which is consistent with findings from other research [14,45]. Meat in supermarkets and retail stores is typically stored in freezers for sale, which may facilitate the growth of this pathogen. Furthermore, cross-contamination during prolonged transportation and preservation can significantly contribute to *L. monocytogenes* contamination. This underscores the significant safety hazard posed by frozen meat and emphasizes the need for improved transportation and preservation conditions to ensure the safety of livestock meat.

The prevalence of *Salmonella* in raw livestock meat was observed at 10.54% in this study, which is higher than the rates reported in some European countries, such as the United Kingdom (2.4%) and Italy (1.57%) [46,47]. Particularly concerning was the detection rate of *Salmonella* in pork, which was 17.60%—significantly higher than that reported in several European countries, including Ireland (0.1%) [48], Scotland (0.3%) [49], and Denmark (3.4%) [50]. In contrast, the prevalence of *Salmonella* in raw livestock meat in China is relatively lower than that reported in some Southeast Asian and Middle Eastern countries. For instance, retail pork samples collected from three provinces in Northern Vietnam exhibited an average *Salmonella*-positive rate of 58.1% out of 671 samples. In Middle Eastern countries, such as Jordan, Saudi Arabia, and Nepal, the detection rates of *Salmonella* in raw animal meat bought from markets and supermarkets were 25.5%, 45%, and 34%, respectively [51,52,53]. Notably, no enteroinvasive *E. coli* or diffusely adherent *E. coli* were detected in any of the samples analyzed. The prevalence of DEC (6.16%) was considerably lower than that reported in Iran (24.76% in raw animal meat) [54].

### 4.2. Serotypes of Salmonella and Pathotypes of DEC

The most common serovar identified in retail raw meat surveyed in China between 2011 and 2016 was *S*. Derby [55]. The majority of documented cases of *Salmonella* infection resulting from the consumption of raw meat are related to pork. Several studies have revealed that *S*. Derby is the most widespread and dominant serotype in pork in China [56,57]. Importantly, source-specific differences in the most frequently detected serovars were observed. *S.* Derby, *S*. Typhimurium, and *S*. Rissen predominated in pork; *S*. Derby, *S*. 1,4,[5],12:i:-, and *S*. London were common in beef; while *S*. Derby, *S*. Typhimurium, and *Salmonella enterica subsp. enterica* serotype Thompson were prevalent in mutton [55]. A global survey noted that *Salmonella* in Asia was mainly dominated by *S*. Derby, whereas *S*. Typhimurium was primarily concentrated in Europe and Oceania [58]. Experimental studies have shown that pigs can carry high concentrations of *S*. Typhimurium asymptomatically in their tonsils, gastrointestinal tract, and mesenteric lymph nodes. These tissues often facilitate the spread of this pathogen to carcasses during slaughter [59,60,61,62,63]. Additionally, the presence of *S*. Typhimurium in the parotid glands poses a significant contamination risk due to potential cross-contamination during incision. This can lead to the dissemination of *Salmonella* throughout the processing line, which may subsequently be isolated from machinery, knives, carcasses, and employee hands [64,65].

In this study, the dominant pathotype of DEC was EAEC, accounting for 84.70% of the isolates. According to GB 4789.6-2016, any detection of the genes *aggR*, *astA*, and *pic* can classify a strain as EAEC. However, only the *aggR* gene is internationally recognized as definitive for EAEC classification [66]. The prevalence of EHEC in pork is higher in South China (13.17%), while it is more prevalent in beef in Northwest China (2.04%) [67]. In certain southern provinces, beef exhibited the highest detection rate (13.32%) among livestock meats, followed by pork at 6.90%, whereas mutton was not detected [68]. A survey of the entire pork production process indicated that 12.8% of 125 healthy pigs tested positive for O157:H7. The prevalence of this pathogen was highest at 86.25% in abattoirs and along the supply chain but dropped to 28.3% in retail sale chains [69].

### 4.3. AMR of Salmonella and DEC

In the United States, *Salmonella* isolates showed no resistance to CIP or NAL, and only a single isolate exhibited resistance to SXT [70]. In contrast, this study identified resistance rates of 51.66%, 20.30%, and 19.19% for SXT, CIP, and NAL among *Salmonella* isolates, respectively. Significant variations in the AMR profiles of *Salmonella* isolates from raw meat have been documented in India and Bangladesh. In India, the predominant resistance was noted against NAL (40.96%), followed by TET at 29.89%. Conversely, Bangladesh reported an alarming TET resistance rate of 81% [71,72]. These discrepancies can likely be attributed to the widespread use of antibiotics in livestock for both disease treatment and growth promotion, coupled with regional differences in antibiotic stewardship practices [20,73].

The prevalence of MDR *Salmonella* isolates in this study was 67.50%, surpassing reports from other countries, such as 48.27% in Iran and 53.84% in Poland [74,75]. Comparatively, reports of *Salmonella* resistance in livestock in the United States are limited; however, an MDR rate of 28.0% was noted in poultry meat from 2008 to 2017 [76]. The issue of MDR in China is critical, highlighting the urgent need for improved surveillance and responsible antibiotic use across all agricultural sectors.

*E. coli* is commonly used to assess AMR in livestock and animal-derived food products. *E. coli* isolates can serve as reservoirs for AMR genes, posing a risk of transmission to humans through various pathways [77,78]. In this study, the resistance rate among DEC isolates reached 84.70%, exceeding the rates reported in other countries, such as 66.07% in Iran and 55.00% in Japan [54,79]. The MDR rates for DEC from dairy cattle (77.8%) and beef cattle (63.6%) [80] were notably higher than those observed in this study, likely due to more extensive antibiotic usage in livestock feeding practices.

To date, many studies have reported main antibiotic-resistant *E. coli* and *Salmonella* from different animal-derived foods in some countries, such as pork, chicken, duck, and fish in China [81,82] and dairy cattle and retail meat in the United States [83,84]. These data denote that foodborne antibiotic resistance is a widespread problem worldwide. China is a major producer and user of antibiotics, not only for the treatment of human bacterial infectious diseases but also for animal husbandry. Several studies in China showed that *Salmonella* isolates from chiken farm exhibit a high frequency of resistance to nalidixic acid [85]. And it isolates from pig farm showed a notable resistance to ampicillin (98.1%) and tetracycline (90.5%) [86]. Due to the long-term, broad, and unreasonable use of antibiotics in animal husbandry, antibiotics remain in farmed animals (including the animal gut and waste). Therefore, there is a serious drug-resistant bacterial burden. These bacteria carry resistance genes, and the resistance of bacteria to drugs is constantly increasing [87]. To ensure human health and animal food safety, new and multidimensional approaches are needed to control bacterial infections for animal production. In recognition of this urgent need, China submitted the National Action Plan in 2016–2020 and updated it for 2022–2025. It focuses on raising public awareness of antimicrobial resistance issues and enhancing knowledge of infection prevention and the rational use of antimicrobial drugs, improving the level of understanding and application among the public firstly. Secondly, it establishes and improves a monitoring and evaluation system for the use of antimicrobial drugs and antimicrobial resistance to provide a basis for scientific decision making. Thirdly, it strengthens scientific research and development in the prevention and control of microbial resistance and promotes the research, development, and application of new antimicrobial drugs, diagnostic tools, vaccines, etc. Finally, it conducts extensive international exchanges and cooperation to jointly address the global challenge of antimicrobial resistance to embracing the One Health Approach to tackle AMR surveillance effectively [88]. The National Nature Science Foundation of China has kicked off several Major Programs that are crucial in the global fight against AMR, safeguarding the health and well-being of all [89].

## 5. Conclusions

The prevalence of three significant foodborne pathogens in raw livestock meat in China was notably high. *L. monocytogenes* was particularly prevalent in frozen and chilled meat products. Furthermore, the rising prevalence of *Salmonella*, especially in warmer regions of southern and western China, raises considerable concerns regarding its presence in pork. It is crucial to implement measures to prevent cross-contamination during transportation and preservation to ensure food safety. Notably, the predominant *Salmonella* serotypes in raw livestock meat appear to be shifting from *S.* Derby to *S.* Typhimurium. Further research through long-term monitoring and analysis is essential to elucidate this trend. Continuous oversight and regulation of antibiotic usage in animal husbandry are vital, as the emergence of MDR in *Salmonella* and DEC presents a significant public health challenge. Ongoing surveillance and control measures are imperative to mitigate these risks.

## Figures and Tables

**Table 1:** Sample size in different provinces and categories of raw livestock meat.

Regions	Provinces	No. of Samples	No. of Samples for Different Categories		
			Pork	Beef	Mutton
Eastern	Shanghai	232	117	63	52
	Jiangsu	212	132	53	27
	Shandong	200	85	74	41
Western	Sichuan	235	135	77	23
	Gansu	165	74	56	35
	Xinjiang	104	30	38	36
Southern	Guangxi	109	46	36	27
	Guizhou	109	63	29	17
	Yunnan	159	78	48	33
Northern	Hebei	215	92	81	42
	Liaoning	130	58	38	34
	Heilongjiang	215	106	56	53
Central	Henan	162	84	38	40
	Hubei	168	68	61	39
	Chongqing	100	48	31	21
Total		2515	1216	779	520

**Table 2:** Prevalence of *L. monocytogenes*, *Salmonella*, and DEC in livestock meat samples in China.

	No. of Samples	*L. monocytogenes*			*Salmonella*			DEC		
		No. of Positive	Prevalence Rate (%)	χ^2^, *p* Value	No. of Positive	Prevalence Rate (%)	χ^2^, *p* Value	No. of Positive	Prevalence Rate (%)	χ^2^, *p* Value
Meat categories										
Pork	1216	110	9.05	χ^2^ = 0.884, *p* > 0.05	214	17.60	χ^2^ = 124.62, *p* < 0.05	96	7.89	χ^2^ = 14.466, *p* < 0.05
Beef	779	66	8.47		32	4.11		29	3.72	
Mutton	520	52	10.00		19	3.65		30	5.77	
Storage states										
Frozen	444	55	12.39	χ^2^ = 43.510, *p* < 0.05	25	5.63	χ^2^ = 15.173, *p* < 0.05	12	2.70	χ^2^ = 12.275, *p* < 0.05
Chill	582	84	14.43		60	10.31		35	6.01	
Fresh	1489	89	5.98		180	12.09		108	7.25	
Regions										
Northern	560	70	12.50	χ^2^ = 24.136, *p* < 0.05	49	8.75	χ^2^ = 21.902, *p* < 0.05	71	12.68	χ^2^ = 89.570, *p* < 0.05
Eastern	644	71	11.02		46	7.14		14	2.17	
Southern	377	25	6.63		56	14.85		12	3.18	
Western	504	25	4.96		68	13.49		13	2.58	
Central	430	37	8.60		46	10.70		45	10.47	
Sampling sites										
Farmer’s market	1220	97	7.95	χ^2^ = 12.466, *p* < 0.05	135	11.07	χ^2^ = 9.011, *p* < 0.05	77	6.31	χ^2^ = 2.400, *p* > 0.05
Supermarket	629	78	12.40		69	10.97		39	6.20	
Retail shop	365	33	9.04		44	12.05		26	7.12	
Catering	301	20	2.21		17	5.65		13	4.32	
Quarters *										
First quarter	154	8	5.19	χ^2^ = 2.126, *p* > 0.05	8	5.19	χ^2^ = 7.86, *p* < 0.05	18	11.69	χ^2^ = 19.746, *p* < 0.05
Second quarter	484	35	7.23		60	12.40		50	10.33	
Third quarter	460	26	5.65		61	13.26		20	4.35	
Fourth quarter	391	20	5.12		51	13.04		20	5.12	
Total	2515	228	9.06		265	10.54		155	6.16	

* There was no difference in the prevalence of pathogenic bacteria between frozen and chilled meat sampled in different quarters; thus, only fresh meat was analyzed.

## Data Availability

The original contributions presented in the study are included in the article, further inquiries can be directed to the corresponding authors.
